# The feasibility of existing JADAS10 cut-off values in clinical practice: a study of data from The Finnish Rheumatology Quality Register

**DOI:** 10.1186/s12969-023-00814-x

**Published:** 2023-04-14

**Authors:** M. Backström, H. Salo, J. Kärki, K. Aalto, K. Rebane, T. Levälampi, M-M. Grönlund, L. Kröger, H. Pohjankoski, M. Hietanen, K. Korkatti, L. Kuusalo, V. Rantalaiho, J. Huhtakangas, H. Relas, T. Pääkkö, E. Löyttyniemi, T. Sokka-Isler, P. Vähäsalo

**Affiliations:** 1 Department of Paediatrics, The Wellbeing Services County of Ostrobothnia, Vaasa, Finland; 2 PEDEGO Research Unit, University of Oulu, Oulu, Finland; 3 Vaasa Central Hospital, U2, Hietalahdenkatu 2-4, 65130, Vaasa, Finland; 4 Knowledge Brokers Department, Finnish Institute for Health and Welfare, Helsinki, Finland; 5 Department of Children and Adolescents, Kanta-Häme Central Hospital, Hämeenlinna, Finland; 6 The Finnish Institute for Welfare and Health, The Finnish Rheumatology Quality Register, Helsinki, Finland; 7 New Children’s Hospital, Pediatric Research Center, Helsinki University Hospital and University of Helsinki, Helsinki, Finland; 8 Department of Paediatrics, Turku University Hospital, Turku, Finland; 9 Department of Children and Adolescents, Kuopio University Hospital, Kuopio, Finland; 10 Department of Children and Adolescents, Päijät-Häme Central Hospital, Lahti, Finland; 11 Department of Paediatrics, Central Ostrobothnia Central Hospital, Kokkola, Finland; 12 Centre for Rheumatology and Clinical Immunology, Division of Medicine, University of Turku and Turku University Hospital, Turku, Finland; 13 Faculty of Medicine and Health Technology, Tampere University, Tampere, Finland; 14 Centre for Rheumatic Diseases, Tampere University Hospital, Tampere, Finland; 15 Centre for Rheumatic Diseases, Kanta-Häme Central Hospital, Hämeenlinna, Finland; 16 Division of Rheumatology, Kuopio University Hospital, Kuopio, Finland; 17 Department of Rheumatology, Inflammation Center, Helsinki University Hospital, and University of Helsinki, Helsinki, Finland; 18 Department of Internal Medicine, Oulu University Hospital, Oulu, Finland; 19 Department of Biostatistics, University of Turku, Turku, Finland; 20 University of Eastern Finland, Kuopio and Central Finland Central Hospital, Jyväskylä, Finland; 21 Department of Paediatrics, Oulu University Hospital, Oulu, Finland; 22 Medical Research Center, Oulu University Hospital and University of Oulu, Oulu, Finland

**Keywords:** Juvenile idiopathic arthritis, Outcome measures, Disease activity

## Abstract

**Background:**

The ten-joint juvenile arthritis disease activity score (JADAS10) is designed to measure the level of disease activity in non-systemic juvenile idiopathic arthritis by providing a single numeric score. The clinical JADAS10 (cJADAS10) is a modification of the JADAS10 that excludes erythrocyte sedimentation rate (ESR). Three different sets of JADAS10/cJADAS10 cut-offs for disease activity states have been published, i.e., the Backström, Consolaro, and Trincianti cut-offs. The objective of this study was to investigate the performance of existing JADAS10 cut-offs in real-life settings using patient data from The Finnish Rheumatology Quality Register (FinRheuma).

**Methods:**

Data were collected from the FinRheuma register. The proportion of patients with an active joint count (AJC) above zero when classified as being in clinically inactive disease (CID) or low disease activity (LDA) groups according to existing JADAS10/cJADAS10 cut-off levels were analyzed.

**Results:**

A significantly larger proportion of the patients classified as being in CID had an AJC > 0 when using the JADAS10/cJADAS10 cut-offs by Trincianti et al. compared to those for the other cut-offs. In the LDA group, a significantly larger proportion of the polyarticular patients (35%/29%) had an AJC of two when Trincianti JADAS10/cJADAS10 cut-offs were used compared with when Backström (11%/10%) and Consolaro (7%/3%) JADAS10/cJADAS10 cut-offs were used.

**Conclusions:**

We found the cut-offs proposed by Consolaro et al. to be the most feasible, since these cut-off levels for CID do not result in the misclassification of active disease as remission, and the proportion of patients with AJC > 1 in the LDA group is lowest using these cut-offs.

## Background

Juvenile idiopathic arthritis (JIA) refers to chronic arthritis that begins before the age of 16 years [1]. Early optimal treatment improves the outcome for this condition [2–6]. The ideal treatment goal is clinically inactive disease (CID) [7, 8], but this is not always possible. It is important to evaluate disease activity on each patient visit and adjust treatment when needed. Accordingly, there have been numerous attempts to develop tools that objectively express the activity of this disease. Disease activity has been divided into different states based on clinical criteria [7–13]. The Wallace preliminary criteria for CID [7] have been expanded to the American College of Rheumatology (ACR) provisional criteria for CID [8], which also embrace the duration of morning stiffness. The Wallace preliminary definition of CID [7] and the ACR provisional criteria of CID [8] have been used consistently in paediatric research. The literature contains several clinical definitions for minimal or low disease activity (LDA), moderate disease activity (MDA), and high disease activity (HDA) [9–13].

Interpreting some of the existing clinical criteria for disease activity levels can be complex and laborious [9–13]. However, the ten-joint count juvenile arthritis disease activity score (JADAS10) [14] and particularly the clinical JADAS10 (cJADAS10) index [15, 16] are more convenient for everyday practice. The JADAS10 is a continuous disease activity score specific to non-systemic onset JIA and comprises four parameters: active joint count (AJC); physician’s global assessment of disease activity (PhGA) using a 10-cm linear visual analogue scale (VAS); parent/patient global assessment of well-being (PaGA) using a 10-cm linear VAS, and erythrocyte sedimentation rate (ESR) [14]. The cJADAS10 is a modification of the JADAS10 without considering ESR [15]. These JADAS10 indexes create uniformity in disease activity evaluation between physicians in clinical work and in research. Nevertheless, assessing the meaning of a single JADAS10 score can be cumbersome. Thus, cut-off values for JADAS10 [12, 13, 17–20] and cJADAS10 [13, 17–20] values have been established for disease activity states (Table 1). However, some disparity exists in the current cut-off sets.

**Table 1:** Ten-joint count juvenile disease activity score (JADAS10) and clinical JADAS10 (cJADAS10) intervals existing in the literatur

	**Oligoarticular disease course**	**Polyarticular disease course**
**Backström et al. **[13, 17, 18]	JADAS10 interval	JADAS10 interval
**CID**	0–0.5	0–0.7
**LDA**	0.6–3.8	0.8–5.1
**MDA**	3.9–6.6	5.2–15.2
**HDA**	> 6.6	> 15,2
**Consolaro et al. **[12, 16, 19]	JADAS10 interval	JADAS10 interval
**CID**	0–1.0	0–1.0
**LDA**	1.1–2.0	1.1–3.8
**MDA**	2.1–4.2	3.9–10.5
**HDA**	> 4.2	> 10.5
**Trincianti et al. **[20]	JADAS10 interval	JADAS10 interval
**CID**	0–1.4	0–2.7
**LDA**	1.5–4.0	2.8–6.0
**MDA**	4.1–13.0	6.1–17.0
**HDA**	> 13.0	> 17.0
**Backström et al. **[13, 17, 18]	cJADAS10 interval	cJADAS10 interval
**CID**	0–0.5	0–0.7
**LDA**	0.6–3.8	0.8–5.0
**MDA**	3.9–6.6	5.1–14.0
**HDA**	> 6.6	> 14.0
**Consolaro et al** [12, 16, 19]	cJADAS10 interval	cJADAS10 interval
**CID**	0–1.0	0–1.0
**LDA**	1.1–1.5	1.1–2.5
**MDA**	1.6–4.0	2.6–8.5
**HDA**	> 4.0	> 8.5
**Trincianti et al** [20]	cJADAS10 interval	cJADAS10 interval
**CID**	0–1.1	0–2.5
**LDA**	1.2–4.0	2.6–5.0
**MDA**	4.1–12	5.1–16.0
**HDA**	> 12	> 16.0

The objective of this study was to investigate the performance of existing JADAS10 cut-off sets, i.e., those by Backström et al. [13, 17, 18], Consolaro et al. [12, 16, 19], and Trincianti et al. [20] using data from real-life patients in The Finnish Rheumatology Quality Register (FinRheuma).

## Methods

We retrospectively collected data from the FinRheuma register for two cohorts. These were:

### Cohort 1

The data from the visits between March 2016 and September 2021 at which non-systemic onset JIA diagnosis according to International League of Associations for Rheumatology (ILAR) criteria [21] was confirmed in disease modifying anti-rheumatic drugs (DMARDs)-naïve patients with non-systemic JIA. The patients had not ever received intra-articular steroid injections at the time of the first registered visit.

### Cohort 2

Non-systemic onset JIA patients aged < 16 years for whom the latest visit was between January 2020 and September 2021.

The two cohorts were chosen in order to get one cohort with many patients with active disease (cohort 1) and one cohort with patients mainly in remission (cohort 2). The selection was done in order to investigate the capacity of the different cut-off values to detect both patient with no or low disease activity as well as high disease activity. Only patients with oligoarthritis, extended oligoarthritis, and rheumatoid factor negative polyarthritis were included in analyses, since the cut-offs according Trincianti et al. [20] are not validated for rheumatoid factor positive polyarthritis, psoriatic arthritis, nor enthesitis-related arthritis. The data on age, gender, ILAR category of JIA [22], AJC, ESR, PhGA, PaGA, and rheumatoid factor (RF) levels were obtained. We used JADAS10/cJADAS10 scores because this is the clinical practice in Finland. For both cohorts, we analysed the distribution of patients in the CID, LDA, MDA, and HDA groups according to existing JADAS10/cJADAS10 cut-off levels. At the latest visit, we also analysed the proportion of patients with AJC > 0 when classified as being in the CID or LDA groups according to existing JADAS10/cJADAS10 cut-off levels. The background data for patients with complete and incomplete data sets were compared in an attempt to detect possible bias arising from the inclusion of only patients with complete data sets.

#### Statistics

Continuous variables are expressed as median and lower (Q1) and upper (Q3) quartiles. Altogether, there were 346 non-systemic JIA patients with a recorded first visit between March 2016 and September 2021 and 1200 non-systemic JIA patients with a recorded latest visit between January 2020 and September 2021 in the FinRheuma register. The differences between clinical characteristics of those who had complete registration of JADAS10 and cJADAS10 and those who had incomplete registration were tested with the Wilcoxon rank sum test for all with continuous variables (e.g. disease duration). When comparing these complete/incomplete patients groups with categorial variables (e.g. proportion of antinuclear antibodies positive/negative) Fisher’s exact test was used. Fisher’s exact test was also used when proportions of active joint count (AJC > 0 and AJC > 1 separately) were compared between different publications. *P*-values lower than 0.05 (two-tailed) were considered to indicate statistical significance. Analyses were performed using SAS System for Windows, version 9.4 (SAS Institute Inc., Cary, NC, USA) and the R Statistical language (version 4.2.1; R Core Team, 2022) on Ubuntu 20.04.5 LTS.

#### Ethics

This study was conducted as a register-based study using data from the FinRheuma register. The quality register is maintained by the Finnish Institute for Health and Welfare (THL), which granted approval for the study.

## Results

### Cohort 1

The FinRheuma register contained 346 DMARD-naïve non-systemic JIA patients who had a registered first visit between March 2016 and September 2021 with a confirmed JIA diagnosis according to ILAR criteria [21]. Of these, 217/346 (63%) and 232/346 (67%) had complete registration of JADAS10 and cJADAS10 parameters. About 2/3 of the patients were girls, and the median (Q1, Q3) age was 8 (4,12) years for patients with both complete and incomplete data. There was a higher proportion of patients with polyarthritis in patients with complete data set (Table 2).

**Table 2:** Clinical characteristics in cohort 1: non-systemic juvenile idiopathic arthritis patients with an incomplete/complete registration of 10-joint count juvenile arthritis disease activity score (JADAS10) and clinical JADAS10 (cJADAS10) parameters at the recorded first visit between March 2016 and September 2021 in The Finnish Rheumatology Quality Register

	JADAS10			cJADAS10		
	Incomplete dataset (*N* = 129)	Complete dataset (*N* = 217)	P	Incomplete dataset (*N* = 114)	Complete dataset (*N* = 232)	*P*
Females n (%)	78 (60%)	147 (68%)	0.209	66 (58%)	159 (69%)	0.056
Age in years, median (Q1,Q3)	7.5 (3.5,11.8)	8.1 (3.7,11.8)	0.606	7.8 (4.0–11.9)	8.1 (3.4,11.5)	0.864
Antinuclear antibodies positive n (% of patients with registered results)	42 (38%)	99 (49%)	0.059	35 (36%)	106 (49%)	0.049
HLAB27 positive n (% of patients with registered results)	33 (30%)	53 (28%)	0.792	29 (30%)	57 (29%)	0.893
Subcategories of JIA						
Oligoarthritis, n (%)	116 (90%)	163 (75%)	< 0.001*	101 (89%)	178 (77%)	0.009*
Polyarthritis, Rheumatoid factor-negative n (%)	13 (10%)	54 (25%)		13 (11%)	54 (23%)	

The difference between groups with continuous variables were tested with Wilcoxon rank sum test and categorial variables with Fisher’s exact test

^*^Fisher´s exact test for the subcategories of JIA

**Fig. 1:**
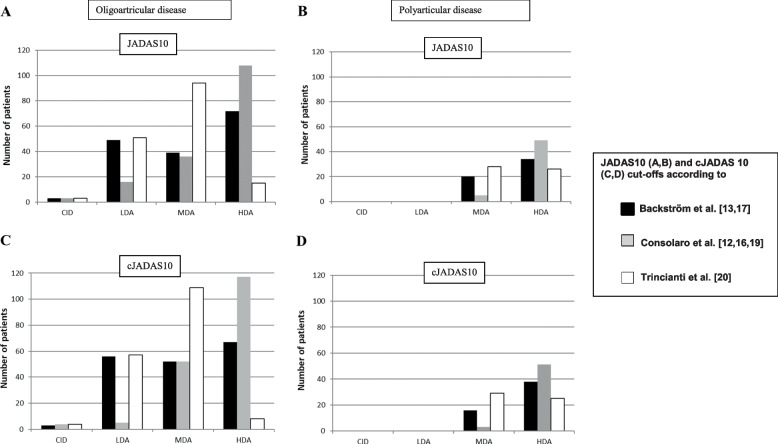
Distribution of the disease activity levels in treatment-naïve oligoartricular (**A**, **C**) and polyartricular (**B**, **D**) patients based on JADAS cut-off values (**A**, **B**) and cJADAS cut-off values (**C**, **D**) according to Backström et al. [13, 17], Consolaro et al. [12, 16, 19] and Trincianti et al. [20]

At the first visit there were divergent distributions of the disease activity states based on existing JADAS10 and cJADAS10 cut-off values [12, 13, 16–20] (Fig. 1). The greatest disparity was seen in the oligoarticular HDA group, where the numbers of patients in the HDA group were 67 (38%), 117 (66%), and 8 (4%,) using the cJADAS cut-offs by Backström et al. [13, 17, 18], Consolaro et al. [12, 16, 19], and Trincianti et al. [20], respectively.

### Cohort 2

There were 1200 non-systemic JIA patients with a recorded latest visit between January 2020 and September 2021 in the FinRheuma register. Of these, 640/954 (53%/80%) patients had a complete registration of JADAS10/cJADAS10 parameters at the latest visit (Table 3). 100/136 (16%/14%) patients with complete registration of JADAS10 /cJADAS10 parameters in cohort 1 were also a part of cohort 2 with complete registration.

**Table 3:** Clinical characteristics in cohort 2: non-systemic juvenile idiopathic arthritis patients with an incomplete/complete registration of 10-joint count juvenile arthritis disease activity score (JADAS10) and clinical JADAS10 (cJADAS10) parameters at the recorded latest visit between January 2020 and September 2021 in The Finnish Rheumatology Quality Register

	JADAS10			cJADAS10		
	Incomplete dataset (*N* = 560)	Complete dataset (*N* = 640)		Incomplete dataset (*N* = 246)	Complete dataset (*N* = 954)	*P*
Females n (%)	372 (66%)	440 (66%)	0.426	168 (68%)	644 (68%)	0.879
Age in years, median (Q1,Q3)	11.7 (8.4,14.2)	11.3 (7.8,14.1)	0.202	11.5 (8.2,14.4)	11.4 (7.3,14.1)	0.480
Disease duration in years, median (Q1,Q3)	4.6 (2.3,8.2)	4.3 (1.9,7.9)	0.063	4.2 (2.3, 8.0)	4.4 (2.1,8.0)	0.697
Antinuclear antibodies positive n (% of patients with registered results)	202 (39%)	276 (45%)	0.047	97 (43%)	381 (42%)	0.706
HLAB27 positive n (% of patients with registered results)	97 (20%)	121 (21%)	0.703	43 (20%)	175 (21%)	0.850
Subcategories of JIA						
Oligoarthritis, persisted n (%)	293 (52%)	318 (50%)	0.658*	113 (46%)	498 (52%)	0.100*
Oligoarthritis, extended n (%)	53 (9%)	63 (10%)		31 (13%)	85 (9%)	
Polyarthritis, Rheumatoid factor-negative n (%)	214 (38%)	259 (40%)		102 (41%)	371 (39%)	

The difference between groups with continuous variables were tested with Wilcoxon rank sum test and categorial variables with Fisher’s exact test

^*^Fisher´s exact test for the subcategories of JIA

**Fig. 2:**
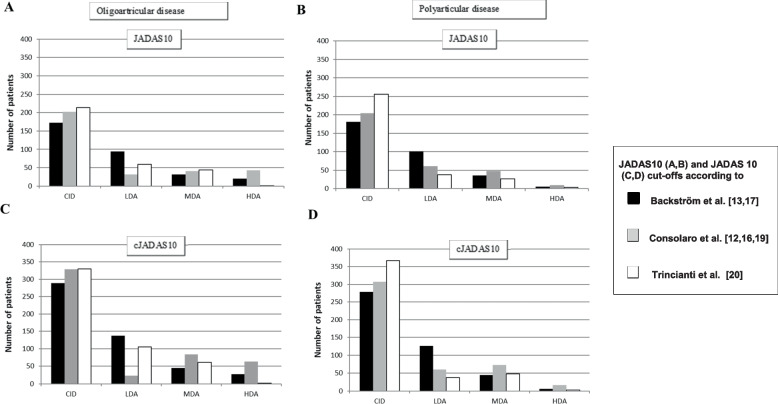
Distribution of the disease activity levels in oligoartricular (**A**, **C**) and polyartricular (**B**, **D**) patients during the latest visit in The Finnish Rheumatology Quality Register based on JADAS cut-off values (**A**, **B**) and cJADAS cut-off values (**C**, **D**) according to Backström et al. [13, 17], Consolaro et al. [12, 16, 19] and Trincianti et al. [20]

At the latest visit, the majority of the patients were in the CID group (Fig. 2). The greatest disparity between the different cut-offs was seen in the cJADAS10 cut-off for CID in polyarticular patients where the number of CID patients were 279, 329, and 367 using the cut-offs Backström et al. [13, 17, 18], Consolaro et al. [12, 16, 19], and Trincianti et al. [1, 2, 20], respectively. In this group, a significantly larger proportion of patients classified as being in CID had an AJC > 0 when using the JADAS10/cJADAS10 cut-offs by Trincianti et al. compared with the other cut-offs (Table 4).

**Table 4:** The proportion of patients at the latest visit with active joint count (AJC) > 0 when in clinically inactive disease (CID) or low disease activity (LDA) according to different existing cut-offs of ten-joint count juvenile arthritis disease activity score (JADAS10) and clinical JADAS10 (cJADAS10)

	Backström et al [13, 17, 18]		Consolaro et al [12, 16, 19]		Trincianti et al [20]		*P*
**JADAS10**							
**Patients in CID**	n	%	n	%	n	%	Fisher´s exact test
Oligoarticular disease course	172		202		214		
AJC > 0	0	0	1	0.5	3	1.4	0.066
Polyarticular disease course	181		204		256		
AJC > 0	0	0	0	0	16	6.2	< 0.001
**Patients in LDA**							
Oligoarticular disease course	94		32		59		
AJC > 0	29	30.9	11	34.3	31	32.5	< 0.001
AJC > 1	5	5.3	0	0	8	13.6	0.004
Polyarticular disease course	101		61		37		
AJC > 0	31	30.7	21	34.4	21	56.8	< 0.001
AJC > 1	11	10.9	4	6.6	13	35.1	< 0.001
**cJADAS10**							
**Patients in CID**							
Oligoarticular disease course	289		329		330		
AJC > 0	0	0	1	0.3	1	0.3	0.242
Polyarticular disease course	279		307		367		
AJC > 0	0	0	0	0	16	4.6	< 0.001
**Patients in LDA**							
Oligoarticular disease course	137		23		105		
AJC > 0	42	30.7	4	17.4	48	45.7	< 0.001
AJC > 1	9	6.6	0	0	14	13.3	0.004
Polyarticular disease course	126		60		38		
AJC > 0	37	29.3	16	26.6	21	55.3	< 0.001
AJC > 1	13	10.3	2	3.3	11	28.9	< 0.001

A marked disparity between the different cut-offs was also seen in the JADAS10 and cJADAS10 cut-offs for LDA in both oligoarticular and polyarticular patients at the latest visit (Fig. 2). In the polyarticular LDA group, the AJC was greater than zero in 30.7%/34.4% of patients when Backström/Consolaro JADAS10 cut-offs were used, compared with 56.8% when Trincianti JADAS10 cut-offs were used (*p* < 0.001). In the LDA group 11%/10% of the polyarticular patients had an AJC of two or more when Backström JADAS10/cJADAS10 cut-offs were used, compared with 7%/3% when Consolaro JADAS10/cJADAS10 and 35%/29% when Trincianti JADAS10/cJADAS10 cut-offs were used (*p* < 0.001) (Table 4).

## Discussion

This Finnish-register-based study showed that, at the latest visit, a small but noticeable proportion of the polyarticular patients in CID and over 50% of the polyarticular patients in LDA had an AJC > 0 according to the latest JADAS10 cut-offs by Trincianti et al. [20]. Furthermore, approximately one third of the polyarticular patients in the LDA group had an AJC of two or more, and a considerably smaller proportion of patients was classified as HDA using JADAS10 and cJADAS10 cut-offs by Trincianti et al., even in the newly diagnosed DMARD-naïve patients. Using the JADAS10 and cJADAS10 cut-offs by Consolaro et al. resulted in the lowest proportion of LDA patients with an AJC of two, both for oligoarticular and polyarticular patients.

The divergence between the studies seeking to find optimal JADAS10 cut-off values might be due to differences in the cohorts as well as the statistical approaches chosen for the analyses. However, above all, the differences are due to divergent classifications of the disease activity states used as a reference. The disease activity states set by Beukelman et al. [10] and used in the studies by Backström et al. [17, 18] are not validated and the HDA definition is set very high. Moreover, the Beukelman criteria [10] state that a patient having a VAS over 2 already has MDA, even if the physician sees no signs of disease activity. This is also the weakness of the disease activity states set by Magni-Manzoni et al. [9] and used in the studies by Consolaro et al. [16, 19], since they state that a patient having a VAS over 2.1 has MDA, even if, again, the physician sees no signs of disease activity. However, the strength of those criteria is that they are objective and can be interpreted in approximately the same way, irrespective of the physician using them. In the latest study on this topic, which was a large multinational study by Trincianti et al. [20], disease activity states were established according to the opinion of the expert, which we suspect is a varying standard. Moreover, these cut-offs were not validated for JIA diagnoses other than those of persisted or extended oligoarthritis and seronegative polyarthritis. They are not intended for seropositive polyarthritis, psoriatic arthritis, nor enthesitis-related JIA.

It is important to include the patient´s perspective in evaluating disease activity but the PaGA parameter in JADAS and cJADAS is prone to rise the JADAS/cJADAS although there are no objective signs of inflammation. It has recently been shown that PaGA correlate better with measures of Health Related Qualify of Life than measures of disease activity [22].

Recommendations for treating Juvenile JIA to target have been formulated by an international task force [23]. Specific treatment targets and guidance on general treatment strategies were described with intention to improve patient care in clinical practice. Despite the ongoing discussion of optimal goals, the main treatment target is preferably CID, and when this is not possible, LDA [23, 24]. Thus, using cut-offs where approximately one third of LDA patients has an AJC of two or more is not optimal. The proportion of LDA patients with an AJC of two was clearly lowest in both oligoarticular and polyarticular patients using the JADAS10 and cJADAS10 cut-offs by Consolaro et al., which is their great advantage.

Another clear benefit of the cut-offs by Consolaro et al. is that the cut-offs for CID are the same regardless of the disease course. The other existing cut-offs require division of the patients in terms of oligoarticular and polyarticular disease courses. Since the oligoarticular and polyarticular disease courses are, rather than different disease entities, spectra of disease activity for different forms of arthritis that come under an umbrella diagnosis of JIA, we think it is both logical and practical to have only one set of JADAS10 cut-offs for disease-activity states, regardless of the oligoarticular or polyarticular disease course.

The strengths of this study are the large number of analysed patients and the inclusion of both newly diagnosed DMARD-naïve patients and patients with a more long-lasting disease course.

A limitation of this study is the lack of an international perspective. It has been shown that physicians in Northern Europe and Finland tend to score PhGA lower than those in other parts of the world [25]. Thus, the results might have been different for a more geographically widespread population.

In conclusion, we found the cut-offs by Consolaro et al. to be the most feasible both in clinical work and in research, since the cut-off levels for CID do not result in patients with AJC ≥ 1 being misclassified as in remission, and the proportion of patients with an AJC of two in the LDA group is the lowest using these cut-offs. A further clear benefit of the Consolaro et al. cut-offs is that the cut-off level is the same for CID in oligoarticular and polyarticular patients.

## Data Availability

Deidentified individual participant data will be made available, in addition to study protocols and the statistical analysis plan. The data will be made available upon publication until December 2032 to researchers who provide a methodologically sound proposal for use in achieving the goals of the approved proposal. Proposals should be submitted to maria.backstrom@ovph.fi.

## Abbreviations

ACR: American College of Rheumatology
AJC: Active joint count
CID: Clinically inactive disease
cJADAS10: Clinical ten-joint count juvenile arthritis disease activity score
DMARDs: Disease modifying anti-rheumatic drugs
ESR: Erythrocyte sedimentation rate
FinRheuma: The Finnish Rheumatologic Quality Register
ILAR: International League of Associations for Rheumatology
JADAS10: The ten-joint count juvenile arthritis disease activity score
JIA: Juvenile idiopathic arthritis
LDA: Low disease activity
MDA: Moderate disease activity
HDA: High disease activity
PaGA: Parent/patient global assessment of well-being
PhGA: Physician’s global assessment of disease activity
RF: Rheumatoid factor
VAS: Visual analogue scale
